# The Post-Acute COVID-19-Vaccination Syndrome in the Light of Pharmacovigilance

**DOI:** 10.3390/vaccines12121378

**Published:** 2024-12-06

**Authors:** Barbara Platschek, Fritz Boege

**Affiliations:** Central Institute of Clinical Chemistry and Laboratory Diagnostics, Medical Faculty, Heinrich Heine University, University Hospital, 40255 Düsseldorf, Germany

**Keywords:** pharmacovigilance, post-acute COVID-19-vaccination syndrome (PACVS), post-acute COVID-19-syndrome (PACS), chronic disease symptoms, product information sheets of SARS-CoV-2 vaccines, European Medical Agency (EMA), EudraVigilance

## Abstract

**Background/Objectives:** Clinical studies show that SARS-CoV-2 vaccination sometimes entails a severe and disabling chronic syndrome termed post-acute-COVID-19-vaccination syndrome (PACVS). PACVS shares similarities with long COVID. Today, PACVS is still not officially recognised as a disease. In contrast, long COVID was registered by health authorities in December 2021. Here, we address possible reasons for that discrepancy. **Methods:** We analyse whether common symptoms of PACVS have been registered by European pharmacovigilance as adverse vaccination reactions and which consequences have been drawn thereof. **Results:** (i) PACVS is distinguished from normal vaccination reactions solely by prolonged duration. (ii) Symptom duration is poorly monitored by post-authorisation pharmacovigilance. (iii) PACVS-specific signals were faithfully recorded by pharmacovigilance systems but have not prompted appropriate reactions of health authorities. (iv) The most widely applied SARS-CoV-2 mRNA-vaccine has been modified after roll-out without renewed phase III evaluation; the modification has increased DNA contaminations suspected to extend the spectrum of adverse events. (v) Crossing of pharmacovigilance data with corresponding estimates of applied vaccine doses suggest a PACVS prevalence of 0.003% in the general population. In contrast, occupational surveillance studies suggest a PACVS prevalence of 0.9% in young and middle-aged persons. **Conclusions:** (a) Denial of official recognition of PACVS is unjustified. (b) PACVS seems to target preferentially young and middle-aged persons. (c) Without official disease recognition, access to public healthcare and welfare services is made difficult for PACVS-affected persons, which creates considerable socio-economic problems. (d) Without official disease recognition, development and evaluation of PACVS therapies is impaired.

## 1. Introduction

Infections with the SARS-CoV-2 virus often trigger an acute disease termed COVID-19, which in rare cases entails a panoply of long-term sequelae summarized as post-acute COVID-19-syndrome (PACS) [1]. Vaccination against the SARS-CoV-2 virus sometimes entails a chronic syndrome that shares many similarities with PACS and has been termed post-acute COVID-19-vaccination syndrome (PACVS) [2]. PACS has been recognized by the World Health Organisation (WHO) in December 2021 by a publication providing a first description and preliminary disease definition [1]. In contrast, PACVS has been perceived by medical science with a delay of three years. To date, PACVS is still not officially recognized as a disease by health authorities worldwide. Here, we address the question of why these two diseases have been handled in such a different manner. We investigate the possibility that PACVS-associated disease symptoms may have escaped official pharmacovigilance systems monitoring undesired side-effects of SARS-CoV-2 vaccines. For that purpose, we compare established PACVS-associated symptoms with undesired side-effects documented in product information sheets of COVID vaccines and other official documents of pharmacovigilance. Furthermore, we address possible contributions of factors other than post-authorization pharmacovigilance to the official disregard of PACVS. Our analysis indicates that many PACVS-associated symptoms have been detected by official pharmacovigilance systems, but these signals have not prompted timely recognition of the disease. Some possible explanations for this oversight are proposed.

## 2. Chronology of Public and Scientific Perception of PACVS

On 21 December 2020, the European Medicines Agency (EMA) granted a conditional marketing authorization to BioNTech (Mainz, Germany) and Pfizer (New York, NY, USA) for their SARS-CoV-2 mRNA-vaccine Comirnaty [3]. Further vaccines based on mRNA-transfection or viral vectors were subsequently introduced. The roll out of these vaccines marks the starting point of one of the biggest vaccination campaigns in the history of Europe, which eventually succeeded in breaking the pandemic caused by the SARS-CoV-2 virus.

About half a year into the vaccination campaign, reports of undesired vaccination side-effects and associated chronic symptoms started to turn up. In May 2021, an online support group published a first summary of self-collected survey data of 508 patients in the USA [4]. In July and September 2021, reports of serious life-altering and long-term symptoms following vaccination were posted by female individuals on the vaccine-critical, controversial platform “Children’s Health Defense” [5,6,7]. In January 2022, a first scientific report of long-term vaccination sequelae was published [8]. In May 2024, the emergence of a chronic syndrome associated with SARS-CoV-2 vaccination was covered by the New York Times [9].

In May 2022, systematic scientific investigations of the novel syndrome associated with SARS-CoV-2 vaccination started with a cohort study carried out by investigators of the National Institute of Health (NIH). This first clinical survey included 23 patients with long-term symptoms following SARS-CoV-2 vaccination. These symptoms encompassed paresthesia, orthostasis, heat intolerance, and palpitations [10]. In 12 of these cases, a peripheral neurological syndrome termed small fibre neuropathy (SFN) was diagnosed by established consensus criteria. The observations of the study conformed to the earlier self-reports posted on public platforms [5,6,7]. The authors of the study concluded that “virtually all preliminary evidence to date supports immune mechanisms” although “enough time has not yet elapsed for the large-scale epidemiological studies necessary to confirm or refute causal relation”. In other words, these authors strongly suspected that paresthesia and other SFN-like symptoms were causally related to SARS-CoV-2 vaccination, but they could not verify that hypothesis by irrefutable proof. These authors also reported that the symptoms could be re-challenged with a follow-up dose of SARS-CoV-2 vaccine in four of the 23 investigated patients. Reactions of European authorities to comparable other adverse events of SARS-CoV-2 vaccination indicate that rechallenging of symptoms in a small number of cases is officially considered a valid indication for causal relationship [11] (pp. 2–3). Thus, rechallenge of SNF-like symptoms in the above study [10] can be taken to provide a strong indication for a causal relationship with SARS-CoV-2-vaccination, although it was only demonstrated in a small subset of study cases.

In March 2023, a systematic review of the published evidence of undesired side-effects of SARS-CoV-2 vaccination discriminated for the first time acute and post-acute (i.e., chronic) SARS-CoV-2 vaccination syndromes and coined the terms ACVS and PACVS, respectively [2]. Most notably, these authors pointed out that PACVS shares many features with post-acute COVID-19-syndrome (PACS, vulgo “long COVID”).

In October 2023, a systematic investigation of 191 individual cases of long-term health conditions following SARS-CoV-2 mRNA-vaccination was published [12]. That clinical cohort study compared alterations of blood markers in PACVS-affected persons with normal vaccination responses of healthy controls and identified certain autoantibodies against G-protein-coupled receptors, which, in the healthy control cohort, were altered following vaccination, whereas humoral immuno-response appeared absent in the PACVS-affected study participants. The authors concluded that PACVS could be due to a lack of immunological adaptation to vaccination and that the above autoantibodies could possibly serve as blood markers for that deficiency.

By the end of 2023 and in 2024, two scientific reports provided an initial aetiological description of the presumed chronic syndrome associated with/following SARS-CoV-2 vaccination. One was a preprint publication of a survey carried out by a consortium of renowned northern American medical institutions, which gauged the disease phenotype based on self-reported long-term symptoms of 241 affected persons [13]. The other report was a peer-reviewed publication attempting a first definition of the PACVS disease phenotype based on self-reports, diagnoses of general practitioners, and alterations of established organ-specific blood markers of 191 PACVS-affected persons [14]. The congruent aetiology of PACVS emerging from these systematic clinical surveys recapitulates many disease-features emerging form earlier case reports and small-scale studies [5,6,7,10], as well as from self-collected survey data of online support groups [4].

The mRNA vaccines by Moderna and BioNtech/Pfizer are the SARS-CoV-2 vaccines most frequently administered in the USA and Europe. Since all available cohort studies and clinical surveys on long-term adverse effects of SARS-CoV-2 vaccination originate from these countries, published data on PACVS are highly biasedtowards mRNA-vaccines. The same applies probably to pharmacovigilance data collected in Europe and the USA. In the published clinical studies and surveys, PACVS cases not linked mRNA vaccinations are mostly associated with administration of the vector vaccines by Astra-Zeneca and Janssen. The prevalence of these cases in the study cohorts (2% [13], 4% [10] and 19% [4]) roughly reflects the employment of the respective vaccines in the USA and Europe. The symptoms triggered by mRNA-vaccines apparently were not different from the symptoms triggered by vector vaccines [4,13] or by sequential combination of the two types of vaccines [12,14]. Virtually no information is publicly available regarding long-term adverse events of any other type of SARS-CoV-2 vaccine (e.g., Novavax, Sinevac).

## 3. Pertinent Questions and Hypotheses

The above chronology of publicly available evidence clearly corroborates the existence of a chronic syndrome associated with, and possibly triggered by, vaccination against the SARS-CoV-2 virus. The available database suggests that the disease can be triggered by various types of SARS-CoV-2 vaccines. However, to date, statistically valid conclusions can only be drawn regarding long-term adverse events following administration of SARS-CoV-2 mRNA-vaccines. Public discussion of that medical problem has apparently been ongoing since summer 2021. Surprisingly, it took the scientific community almost three more years to elaborate and publish a first etiological description of that newly discovered syndrome [13,14]. Moreover, PACVS is still not officially recognized as a disease. In other words, PACS and PACVS have almost simultaneously appeared as widespread health conditions. PACS has been timely investigated by the scientific community and recognized by the WHO as a novel disease. In contrast, PACVS has been scientifically investigated with a delay of several years and is still not recognized as a vaccination-associated disease or syndrome by health authorities.

Here, we try to elucidate what has caused the striking delay of scientific investigation of PACVS as compared to PACS and why PACVS is still lacking official recognition as a vaccination-associated disease. The most obvious explanation seems, that the time frame of pharmacovigilance of COVID-19-vaccines was not adjusted to long-term undesired effects. Thus, alarming signals of chronic health conditions following SARS-CoV-2 vaccination [4,5,6,7,8,9,10,12,13,14] could have escaped official pharmacovigilance systems because these were focussed on monitoring acute adverse effects during the initial vaccination campaign [15]. Conversely, one could doubt the validity of public reports [4,5,6,7,8,9] and of small-scale scientific studies [10], which postulate the existence of long-term disease symptoms following SARS-CoV-2 vaccination because these findings were not detected/recorded by official pharmacovigilance systems. In summary, two explanatory hypotheses can be tested:

• PACVS has been missed by the currently implemented pharmacovigilance systems because these were maladapted to long-term adverse events.
• Signals of chronic adverse side-effects of COVID-19-vaccines have faithfully been recorded by currently implemented pharmacovigilance systems, but appropriate conclusions were not drawn from these recordings.

## 4. Listing of Common PACVS Symptoms as Adverse Events of SARS-CoV-2 Vaccines

To address the above hypotheses, we first compared PACVS-associated symptoms as delineated by published cohort studies [13,14] with adverse events listed in the most recent product information sheets of the various SARS-CoV-2 vaccines provided to the public by the EMA [16,17,18,19,20,21]. Comparisons of 107 of 110 PACVS-associated symptoms identified by a clinical cohort study [14] are summarized in Table S1. Symptoms that were excluded from the analysis due to insufficient clarity of denomination are marked in Table S1 as “no adverse event detection possible”. Of the PACVS-associated symptoms or diagnoses that could be addressed in the analysis, more than half (67/107) exhibited unambiguous correlations to comparable listings of adverse events in the vaccines’ product information sheets. Moreover, the symptoms most frequently exhibited by PACVS-afflicted study participants (prevalence 58–85%) [14] were in majority (23/30) also explicitly listed in the vaccines’ product information sheets (Table 1 and Table S1, lines 1–30).

Taken together, the adverse drug reactions listed in data sheets provide an astonishingly complete representation of the symptoms commonly observed in PACVS [13,14]. The PACVS-relevant symptoms listed as known adverse events fall into three groups: (i) the common reactogenicity adverse events of fatigue, myalgia, headache, arthralgia, dia–rhea, vomiting, chills, and fever; (ii) neurological symptoms, most notably paresthesia/hypoesthesia; (iii) cardiovascular reactions of dizziness, palpitations, tachycardia, alterations of blood pressure. All three groups are classified in the product information sheets as transient and harmless, and in majority, are blamed on common reactogenicity or anxiety-related reactions to extreme stress, e.g., due to hyperventilation. However, these very same adverse events provide a fairly comprehensive summary of the severe long-term symptoms commonly exhibited by persons suffering from PACVS (Table 1, compare 18 of the 30 most common symptoms, lines 1–3, 5, 6, 8, 12–14, 16, 17, 21–23, 25, 27, 28, 30).

The apparent correlation between the aetiology of PACVS as defined in clinical cohort studies [12,13] and presumably harmless adverse effects of the various SARS-CoV-2 vaccines documented in product information sheets favour the second one of the above two hypotheses: Most of the symptoms commonly associated with PACVS have indeed been faithfully monitored and recorded by European pharmacovigilance systems. However, these signals have not entailed detection/definition of PACVS as a novel vaccination-associated disease by the health authorities. In order to explore potential explanations for that lack of perception, we address subsequently in detail how the recordings of above PACVS-relevant adverse events of SARS-CoV-2 vaccination were evaluated by the EMA. This analysis will be structured according to the above three groups of PACVS-related adverse effects.

## 5. Overlap of the PACVS Symptoms with Common Reactogenicity Adverse Events

The vast majority of PACVS-associated symptoms identified by scientific cohort studies correspond to adverse events listed in the product information sheets of the vaccines with a very high incidence. Thus, 54/67 of the PACVS-conforming adverse events listed in Table S1 correspond to adverse events listed in the respective product information sheets as having a known frequency, with 89% (48/54) thereof specified as “uncommon” (23/54) or even “very common” (25/54). These highly frequent adverse events include 19 of the 30 most common PACVS-associated symptoms identified by scientific studies (Table 1), which can all be correlated to adverse events specified as “uncommon” (10/19) or “very common” (9/19).

The PACVS-associated symptoms recorded by pharmacovigilance systems as highly frequent adverse events exhibit a substantial overlap with systemic reactogenicity adverse events detected in the initial clinical phase III study of the vaccine Comirnaty [22], comprising fatigue, muscle pain, headache, joint pain, diarrhoea, vomiting, chills, and fever (see: [22], Table 2B). All of these symptoms have a considerable prevalence in PACVS cohorts (vomiting 13%, fever 20%, diarrhoea and chills 40%, joint pain 61%, headache 63%, muscle pain and fatigue > 80%) (Table 2 and Table S1, lines 1, 3, 8, 22, 25, 50, 51, 87, and 100, respectively). In the Comirnaty phase III study [22], the reactogenicity adverse events were mostly evolving within the first two days after application of the vaccine and subsided shortly thereafter. The chronic symptoms of PACVS were similarly reported to have started within the first few days after vaccine administration [4,13]. This observation suggests that PACVS develops by pathological persistence of a normally transient (and harmless) vaccination reaction. Fitting that conclusion, PACVS-afflicted individuals fail to exhibit vaccination-induced alterations of blood markers, which, in healthy controls, possibly reflect successful coping with vaccination [12]. Thus, PACVS imposes as prolonged version of the normal vaccination reaction, whereas PACS (long COVID) differs significantly from the acute disease from which it emerges [2].

In summary, many symptoms of PACVS are identical to reactogenicity adverse events registered in phase III studies or by post-authorization surveillance of SARS-CoV-2 vaccines. In the product information of the vaccines, these adverse events are classified as acute reactions evolving during the first days after vaccine administration [4,13]. Since PACVS apparently results from pathological persistence of these normally transient vaccine reactions, it could have escaped official perception, because the duration of above adverse events was not systematically monitored by pharmacovigilance systems.

## 6. Symptom Duration of PACVS-Related Reactogenicity Adverse Events

The most comprehensive data on the duration of PACVS-associated symptoms is provided by an on-line survey carried out on 508 patients suffering from persistent neurological symptoms after receiving the SARS-CoV-2 vaccine in the United States [4]. A total of 35% of these participants reported improvement of symptoms within the first six months. The rest reported persistence or aggravation of symptoms during a follow-up period of nine month. Thus, in a third of PACVS cases, the symptoms subsided within half a year, while otherwise, they persisted or became even worse during nine months. In keeping with that timeframe, subsequent clinical cohort studies of PACVS used as inclusion criterion symptom persistence after the last round of vaccination for five months or more [12,13,14].

In comparison to the available clinical surveys [4,12,13,14], publicly available records of EudraVigilance (the pharmacovigilance system operated by the EMA for monitoring adverse drug events post-authorization) provide less precise information on symptom duration. In the publicly available part of that database adverse vaccination-reaction are classified as “recovered”, “recovering”, or “not recovered” at the time of the last observation [23], as prescribed by the Access Policy of EudraVigilance [24] (p. 56). However, results of systematic follow-up investigations of these cases are unavailable in the database or not disclosed to the public [4]. Minimal duration of symptoms can also not be derived from the time-lapse between vaccination and corresponding report of adverse event because these time marks are stored in the non-disclosed part of the EudraVigilance database [24] (pp. 37, 62). Thus, it is impossible to determine whether cases registered as “still recovering” or “not recovered” presented with prolonged symptoms matching the clinical phenotype of PACVS [4,12,13,14].

To sum up, PACVS is characterized by prolonged duration of symptoms overlapping with adverse events frequently registered by pharmacovigilance [4,13] and with reactogenicity adverse events reported in phase III studies [22]. A representative documentation of the precise time frame of these adverse vaccination reactions is not available, although official records display a few cases, in which amelioration or even disappearance of these symptoms within the first weeks or months has been reported [4]. The striking similarity of the adverse events (of undetermined duration) recorded in the EudraVigilance database, and the prolonged symptoms delineated by clinical studies of PACVS suggest that it is only the duration of symptoms that discriminates PACVS from normal, transient, and short-term vaccination reactions. Incidentally, this notion is supported by a surveillance study of 877 Czech workers [25]. In total, 814 of these exhibited adverse events of SARS-CoV-2 vaccination. The main symptoms were pain at the puncture site, myalgia, headaches, and fatigue. These symptoms were similar irrespective of their duration. Duration was specified as immediate (up to three days, 653 cases), intermediate (up to one month, 143 cases), or long-term (more than one month, 11 cases). Although that study was terminated after one month (and thus does not cover the time-frame relevant for PACVS), it corroborates the notion that long- and short-term vaccination sequelae share the same symptoms.

## 7. PACVS-Specific Neurological Dysfunctions as Short-Term Adverse Effects

In addition to the frequent symptoms of common reactogenicity adverse events discussed above, clinical studies have also identified a variety of common PACVS-associated symptoms that exhibit a much higher disease specificity. Two prominent examples are paresthesia and hypoesthesia, which have a high prevalence in PACVS (80% and 62%, respectively, see: Table 1, lines 6 and 23). These symptoms occur otherwise with high incidence in peripheral neuropathy and impaired peripheral blood circulation, but they are rare in other diseases or normal life. Thus, in the context of SARS-CoV-2-vaccination, paresthesia and hypoesthesia can be considered as disease-specific symptoms if the few and well-defined other causes can be excluded.

Until August 2021, EudraVigilance has recorded 21,793 cases of paresthesia/hypoesthesia, which were reported spontaneously in conjunction with application of the most common vaccine Comirnaty. Consequently, the EMA has included these symptoms as frequent adverse reaction in the post-authorization documentation of Comirnaty [26,27] and mandated that they should be added as established side effects to the product information of that vaccine [26] (p. 3). Paresthesia/hypoesthesia were first classified as “anxiety-related reactions” [16]. In January 2022, the EMA realized that these neurological symptoms were frequently (40% of cases) reported in conjunction with reactogenicity adverse events but in the absence of anxiety-related reactions. Consequently, reclassification as adverse events independent of stress has been mandated [27] (p. 50, application number II/0080). However, paresthesia/hypoesthesia was not reclassified as an adverse event of special interest, and the specific link to chronic vaccination sequelae was not perceived.

To understand this oversight, one must consider how the duration of these symptoms was judged: Before mandating the addition of paresthesia/hypoesthesia as adverse events to the product information of Comirnaty, the EMA analysed symptom duration. It came to the conclusion that in 70% of the cases, the two symptoms persisted for two days or less [27]. It was probably this finding which prompted their initial listing as harmless and transient adverse events. However, that investigation was strongly biassed towards short-term events because it was restricted to those cases (15%) for which duration was recorded as “known”. All other cases (85%) were excluded from the analysis. It stands to reason that the analysis excluded all cases of PACVS, which were ongoing and therefore not registered as having a “known” duration.

One can compare the (presumably biased) results of symptom duration of the EMA with the outcomes of reported events of paresthesia/hypoesthesia recorded by EudraVigilance for Comirnaty until August 2024 [24]. In that database, 56.6% of all cases of paresthesia and 53.5% of all cases of hypoesthesia are reported as either “recovering” or “not recovered”, which seems to indicate that the symptoms could have persisted beyond the last recorded observation. However, the apparent discrepancy between these records and the results of the symptom duration study is not addressed/discussed in the EMA’s final statement [27], suggesting that it has escaped notice. No documentation is available regarding efforts of the EMA to derive follow-up information on symptom duration, e. g., by screening the time lapses between vaccination and the report of adverse reactions, although these time stamps are available in the EudraVigilance database [23].

Interestingly, the above approach has recently been used by The Netherlands Pharmacovigilance Centre (Lareb) [28]. Adverse events of SARS-CoV-2 vaccination persisting for six months or more were detected in the registry by screening for ongoing reactions with extended time lapses between the start of the adverse event and the date of reporting. Long-term adverse events following SARS-CoV-2 vaccination thus detected in the registry share many features with PACS (long COVID). It has therefore been concluded in the report that COVID-19 cannot be ruled out as a possible cause of the long-term symptoms [28]. The report does not comment on the striking similarity between the long-lasting symptoms (addressed as “long COVID-like”) and short-term reactogenicity adverse events of SARS-CoV-2 vaccination recorded in the registry. However, that similarity seems to argue in favour of vaccination being the cause of the symptoms, rather than COVID-19.

In summary, persistent paresthesia and hypoesthesia are specific and common symptoms of PACVS. These known adverse events of Comirnaty can persist for many months, which has apparently escaped the EMA’s scrutiny. This oversight is probably due to (i) the restricted time frame, within which data were selected for initial analysis of symptom-duration, (ii) incomplete re-evaluation of the initial findings in the light of subsequent EudraVigilance recordings, and (iii) assumption of causes other than vaccination.

## 8. PACVS-Specific Cardiovascular Symptoms Listed as Anxiety-Related Adverse Effects

In PACVS, the cardiovascular symptoms of dizziness, palpitations, tachycardia, and alterations in blood pressure have a prevalence of up to 80% (Table 3 and Table S1, lines 5, 13, 17, 28, 30, 31, 47, 54, 62 and 76). In the product information of SARS-CoV-2 vaccines, these symptoms are also listed, albeit as normal transient vaccination reactions related to stress and anxiety. It is entirely unclear what has prompted this rather bland classification, since publicly available documents contain no indication of any investigation that would have justified to consider these symptoms as normal, transient and harmless responses to vaccination. Quite the contrary: the product information of the SARS-CoV-2 peptide vaccine Novavax lists hypertension as transient adverse event (unrelated to anxiety) [20], and one case of tachycardia has been documented as serious adverse event related to vaccination in the clinical phase III study of Comirnaty [22]. Along the same lines, the early NIH-based cohort study on long-term adverse reactions to SARS-CoV-2 vaccination reported cardiovascular symptoms in the context of dysautonomia and SFN, which in some cases even could be rechallenged by renewed vaccination [10]. These study data are corroborated by a recent case report on chronic myopericarditis, dysautonomia, and neurological disorders following SARS-CoV-2 vaccination [29]. In summary, these data argue against the classification of vaccination-related cardiovascular symptoms as being a “normal” and “transient” reaction related to stress and anxiety. Since the NIH-based study was conducted between January and September 2021 ([10], p. 5) under the purview of the national health surveillance system of the USA, these data should have been available to the EMA. And yet, none of the above information was taken into account when rating the cardiovascular symptoms as anxiety-related vaccination responses.

## 9. Post-Authorization Alterations of the Manufacturing Process of mRNA-Vaccines

Misapprehension of the duration of adverse reaction to SARS-CoV-2 vaccination as outlined in the previous chapters is just one possible explanation of why PACVS has been overlooked by health authorities worldwide. Another possibility is that crucial properties of the vaccines have been altered after authorization and that these alterations possibly have increased the propensity of the vaccines to induce PACVS. One example demonstrating this possibility is the link between mRNA vaccines and the PACVS-specific symptoms of paresthesia/hypoesthesia, cardiovascular symptoms, and reactogenicity adverse events. In the case of Comirnaty, paresthesia/hypoesthesia and the cardiovascular symptoms were not seen during the phase III study. However, they appeared later in the course of post-authorization monitoring. Interestingly, the manufacturing process of Comirnaty had been changed between these two sets of observations, namely from process 1 (generating the test-material for the clinical trials) to process 2 (generating the commercial product employed in the vaccination campaign) [30] (p. 18). The alternative production process 2 was approved about two months after the start of the European vaccination campaign, in which the altered product had already been widely used. The approval document states that the different production route leads to a slightly different end-product containing a higher amount of residual DNA. To accommodate that alteration without a renewed validation/authorization cycle, specification of a limit of DNA content “considered suitably low” was added to the approval document [30] (p. 21).

Meanwhile, it is doubted that the DNA present in the altered vaccine is indeed as harmless as initially assumed [31,32,33,34]. It is argued that DNA is a much more stable and durable molecule than mRNA, and that the nanoparticles present in mRNA-vaccines stabilize the DNA, thereby creating compounds close to the reagents used in experimental vector-free gene transfer [32,35]. It has been demonstrated that the amount of DNA impurities in the vaccines is correlated to serious adverse events of vaccination [31]. And it has been proposed that these DNA impurities could provoke sustained autoimmune reactions, promote cancer, or induce cardiac arrest [32].

Moreover, the specification of the limit of DNA impurities in the vaccines is open to discussion. On the one hand, the specified quantification process seems to be indirect and fallible [33]. More importantly, the limit for DNA impurities is defined as mass concentration [ng/dose] without regarding the number of DNA fragments from which that mass is constituted. Therefore, enzymatic DNA cleavage during the production process leaves behind impurities of plasmid DNA consisting of a very high number of very small DNA fragments that, in mass sum, remain below specification limit. It is assumed that a single vaccine dose can contain more than 10^11^ such oligonucleotides [31,32].

Up to now, national health authorities have not yielded to the above criticism. They insist that “residual amount of plasmid DNA is present in small amounts that are considered harmless below a threshold specified in the marketing authorisation. To date, there is no evidence to suggest that any adverse events could be associated with residual DNA levels in authorised COVID-19-mRNA vaccines” [34]. This point of view probably applies to the vast majority of vaccinations, which do not exhibit persistent adverse reactions. However, PACVS may well pose an entirely different problem, of which health authorities currently are not aware. It has been demonstrated that the vast majority of PACVS-afflicted individuals exhibit extremely high levels of circulating interleukins 6 and 8 accompanied by abnormally low levels of free tri-iodine thyroxine (fT3) [14]. This constellation is typically seen in intensive care and occurs also in PACS (long COVID). It is considered to indicate a high level of systemic inflammation conforming to sustained autoimmune reactions possibly induced by DNA impurities in the vaccine [32]. Thus, it may well be worth the while for health authorities to entertain the idea that DNA impurities of SARS-CoV-21 vaccines have the potential to induce prolonged systemic inflammation, which fortunately happens not very often (see Section 10). However, if it happens, it may engender a severe chronic syndrome named PACVS.

Residual DNA, as a possible cause for PACVS, could explain why long-lasting adverse events have been observed during the vaccination campaign but not during the clinical phase III studies of the mRNA vaccines. However, this theory cannot explain why PACVS has occasionally also been observed after vaccination with vector vaccines [4,10,13]. So far, the available studies have, in majority, addressed PACVS triggered by mRNA vaccines. Based on the comparable small cohort sizes and the very minor admixture cases triggered by vector vaccines, vaccine-type-specific differences in the clinical presentation of PACVS could probably not be detected with statistical significance. Since PACVS mainly presents as systemic inflammation, it possibly constitutes a common final pathway of diverse pathogenic cascades triggered by diverse types of SARS-CoV-2 vaccines. Conversely, a common denominator, such as the persistence of the spike S1 protein [36], could be the single cause of the uniform pathology triggered by several types of SARS-CoV-2 vaccines as well as by SARS-CoV-2 infection (long COVID). However, neither a common final pathway nor a common denominator can explain why long-term adverse symptoms have not been registered during the phase III clinical trials of the vaccines. Thus, further studies are needed to assess the propensity of the various types of SARS-CoV-2 vaccines to trigger PACVS and to determine the clinical phenotype of PACVS associated with the various types of vaccines. Ultimately, such studies may also provide insight into the pathogenic mechanisms linking SARS-CoV-2 vaccination to PACVS.

## 10. Bias of PACVS Prevalence Towards Young and Middle-Aged Persons

Since PACVS has not been recognised as a disease entity, its prevalence is currently ill-defined. Registries of self-help groups and on-line surveys [4,5,8,9] as well as unofficial statements of informed members of health authorities [9] suggest, that a considerable number of vaccinated persons are affected. Obtaining a more precise estimate of the prevalence is not a simple task, because it is only the prolonged duration of symptoms that distinguishes PACVS from a normal vaccine reaction (see Section 6). Thus, the issue is hinged on the question which symptom duration can be considered as normal as opposed to uncommonly prolonged.

That said, the overall prevalence of PACVS in the vaccinated population can be gauged from the number of cases recorded by post-authorisation pharmacovigilance as being “recovering” or having “not recovered” from PACVS-specific neurological symptoms following SARS-CoV-2 vaccination. By August 2021, roughly 28,000 cases exhibiting such symptoms have been recorded in conjunction with Comirnaty [26,27]. A later statistic suggests that about 55% of these cases can be considered to be ongoing [24]. And a large online survey suggests that one-third of ongoing cases probably recover within six months [4]. Taking all these factors into account, one can assume that by August 2021, up to 10,000 persons across Europe could have been suffering from persistent debilitating symptoms related to vaccination with Comirnaty. Putting that figure in relation to the estimated 1.2 billion doses of vaccine administered world-wide at that time [26], this leads to an estimate of PACVS prevalence linked to Comirnaty in the range of 0.003%. Estimates of PACVS incidence in a similar order of magnitude can be found in the literature [2,12].

However, above calculations on global PACVS prevalence need to be taken with a grain of salt, given the uncertainties regarding the number of vaccine doses administered per person, the fraction of vaccine doses administered in Europe, and the coverage of adverse events in Europe by the EudraVigilance database.

A much more disquieting figure of PACVS prevalence can be extracted from studies monitoring the duration of adverse symptoms following SRAS-Cov-2 vaccination in selected cohorts. A convincing example is the surveillance study of 877 Czech workers [25]. Following SARS-CoV-2 vaccination, 1.4% of the participants suffered for more than one month from PACVS-like adverse events. Only one third of these cases can be assumed to have recovered within five months thereafter [4]. The remaining 0.9% possibly reflect the prevalence of persistent debilitating PACVS in a European working population, which implies that PACVS prevalence could be manyfold higher in the working population than in the general population. The latter notion is also indicated by the age-distribution of PACVS-affected persons in clinical cohorts and online surveys, which exhibits a significant bias towards young and middle-aged adults [4,5,8,9,12,13,14].

## 11. Conclusions

### 11.1. Salient Findings

Our analysis addresses the question why PACVS is still not officially recognised as a novel disease more than three years after it first appeared. In principle, national health authorities should have been capable of doing so, since they were able to recognise and define the temporarily coincident and phenotypically similar disease PACS (long COVID) within little over a year after it first appeared. We suspect that PASCVS could have been overlooked due to an astonishing combination of blind spots in European pharmacovigilance: (i) Pharmacovigilance systems were sub-optimally adjusted to monitor and follow-up on pathological vaccine reactions that are distinguished from normal reactions only by an unusually long duration. (ii) The limited set of PACVS-relevant signals that has been faithfully recorded by post-authorization monitoring by EudraVigilance has not prompted appropriate official reactions by the EMA. (iii) Composition of the most widely used SARS-CoV-2 vaccine Comirnaty has been modified during the vaccination campaign without renewed phase III evaluation, and these untested modifications have possibly engendered the altered spectrum of adverse events entailing PACVS.

### 11.2. Limitations

Our argumentation is limited by several inevitable factors: (i) We could only analyse published data and official sources open to the public. (ii) Deliberations and decisions of the EMA had, in most cases, to be deduced from short text sections in the pharmacovigilance and authorization documents, which may have been misinterpreted out of context. (iii) Crucial parts of the pharmacovigilance database are not accessible to the public due to data protection and privacy rules. (iv) Our analysis is, to some degree, based on independent scientific investigations and surveys that included only a limited number of persons. (v) We focus on studies and pharmacovigilance data originating from USA and Europe, which are highly biasedtowards SARS-CoV-2 mRNA vaccines. In conclusion, it would be sensible to corroborate our data and render our analysis more reliable by replicating the investigations based on large-scale pharmacovigilance data, also covering the administration of SARS-CoV-2 vaccines uncommon in USA and Europe. However, due to data privacy, such studies would have to be internal investigations of the national authorities themselves.

### 11.3. Political Aspects

Corroborative investigations by national health authorities could allow for countering the widespread suspicion that overlooking PACVS has been a political convenience. Shortcomings of pharmacovigilance, lack of corresponding reactions by national health authorities, and unvalidated changes in vaccine composition may have contributed to overlooking PACVS. However, one cannot ignore that PACVS has been highly inconvenient in political terms. During the pandemic, health authorities were acting under great pressure. They had to launch vaccination campaigns on a vast scale and to render these campaigns a rapid success. A prerequisite for that endeavour has been the firm assurance of the general public that the unprecedented pace of development, authorization, and roll-out of SARS-CoV-2 vaccines has not encompassed any compromises regarding safety. It is understandable that an event such as PACVS has been highly unwelcome because it casts potential doubt on the robustness of pharmaco-authorisation and pharmacovigilance of SARS-CoV-2 vaccines. Perhaps it was for that reason that PACVS has not been investigated as eagerly as PACS, and that health authorities and public health services still adhere to that position [37]. However, by now, since the SARS-CoV-2 pandemic is ending and as public healthcare returns to a normal mode, it seems highly imprudent to continue to ignore a chronic health condition that possibly concerns (and incapacitates) a substantial fraction of the young and middle-aged adult population. The time may have come to revise the official position regarding PACVS. Such a revision also seems sensible, since the issue is meanwhile addressed by independent scientific investigation [2,8,9,10,12,13,14,25,38]. To ignore that growing body of published evidence will become increasingly difficult.

### 11.4. Social Aspects

Lack of official disease recognition has serious adverse consequences for the private life of PACVS-affected persons. Without an established diagnosis, their chronic health condition is often not accepted as a probable cause for disability nor as valid justification for the inability to continue professional occupation or educational carrier. These patients are often forced to conduct legal processes in order to obtain proper documentation of their health status. They are denied proper healthcare because medical professionals not knowing the disease dismiss their case altogether or misinterpret their health condition as a psychosomatic illness [39,40,41,42,43,44]. Health insurance companies not knowing the disease deny refunding of PACVS-associated healthcare expenses. These mechanisms act in concert and often lead to rapid socio-economic decline of PACVS-affected persons. Given that these patients are in majority young or middle-aged, PACVS inadvertently engenders a growing load of long-term social problems. For that reason alone, it seems mandatory to officially recognise PACVS and enable public healthcare and welfare services for the affected.

### 11.5. Therapy Concepts and Confounding Comorbidities

The example of PACS (long COVID) demonstrates that timely recognition of a new disease by health authorities provides a crucial driving force for fast scientific progress on diagnosis [45,46,47] and therapy [48,49]. Conversely, the example of PACVS demonstrates that denial of that recognition delays such medical progress. So far, only a handful of publications have addressed the clinical phenotype, pathogenic mechanisms, and diagnostic markers of PACVS [12,13,14,31,33,44]. Most notably, very little is known about options of PACVS therapy, and controlled therapy studies are not available.

Currently, PACVS patients are mostly subjected to symptomatic treatment by family doctors and experimental self-therapy. The survey on 241 PACVS patients [13] gives an impressive picture of that scenario. It reports that a total number of 209 individual therapies were tested. In the median, each patient had undergone 20 individual treatments. Only a comparatively small fraction of treatments encompassed prescription drugs including oral steroids (48%), gabapentin (25%), low-dose naltrexone (20%), ivermectin (18%), propranolol (11%), and bronchodilators (11%). More than 500 additional treatments were tried out by the patients in an unguided manner. The latter included intake of probiotics, vitamins, and supplements (85%–100%); limitation of exercise or exertion (51%); increases in salt intake or hydration (44%); and intermittent fasting (39%). The wide spectrum of pharmacological targets and the sheer number of compounds and physical therapies tried out in seemingly aleatory manner demonstrates an urgent need for PACVS therapy.

The demand for controlled therapy studies implicated by the above scenario is difficult to meet. Without proper disease criteria, it is difficult to obtain funding and ethics approval for therapy studies and controlled trials of off-label therapies. More importantly, without proper disease criteria, it is difficult to identify bona fide cases of PACVS suitable for such trials. On the one hand, the disease phenotype of PACVS is heterogeneous and possibly encompasses more than one clinical entity [14]. On the other hand, bona fide long-term sequelae of SARS-CoV-2 vaccination are confounded by co-morbidities and pre-existing diseases, which become exacerbated or reactivated by vaccination. The latter includes Guillain–Barré syndrome, IgA nephropathy, lupus nephritis, and various other auto-immune diseases [29,50,51,52,53]. These co-morbidities are not uncommon. More than 80% of the candidates applying for participation in a clinical cohort study on PACVS had to be excluded due to confounding co-morbidities [12].

Another major confounding factor is intermittent infections with SARS-CoV-2 virus, which possibly lead to an overlay of PACVS and PACS. Incidentally, attempts of PACVS-affected persons to obtaining legal endorsement of their disease have been ruled out of court on the grounds that it could not be safely excluded that their symptoms were due to PACS. Incidentally, this argument has been reiterated in a recent report by Lareb on Long-COVID-like symptoms following COVID-19-vaccination [28]. While PACS and PACVS present in part with similar symptoms, it is improbable that they will respond to the same therapy. The current belief holds that PACS can be due to persistence of the SARS-CoV-2 virus [46], while that is a highly improbable cause of PACVS. In summary, it seems important to further investigate similarities and differences in PACS and PACVS, e.g., by comparing published cohort studies on the two diseases.

## Figures and Tables

**Table 1:** Most common PACVS-associated clinical symptoms ^1^ and corresponding adverse events listed in the vaccines’ product information.

No ^2^	PACVS-Associated Symptoms ^2^	Prevalence ^2^	Monitored Adverse Events According to the Vaccine’s Product Information ^3^					
			Biontech/Pfizer	Moderna	AstraZeneca	Janssen	Novavax	Valneva
1	exhaustion	85%	fatigue, v.c.	fatigue, sleepiness, v.c.	fatigue, v.c.	fatigue, v.c.	fatigue, v.c.	fatigue, v.c.
2	debility	84%	asthenia, uc		asthenia, c.	asthenia, muscular weakness, uc.		
3	muscle pain	81%	myalgia, v.c.	myalgia, v.c.	myalgia, v.c.	myalgia, v.c.	myalgia, v.c.	myalgia, muscle spasms, v.c., uc.
4	unrestful sleep	81%	insomnia, uc					
5	dizziness	80%	dizziness, uc	dizziness, uc.	dizziness, c.	dizziness, uc.		dizziness, uc.
6	tingling/prickling/paresthesia	80%	paresthesia, n.k.	paresthesia, r.	paresthesia, uc.	paresthesia, r.	paresthesia, n.k.	paresthesia, uc.
7	impairment of mental focussing	79%						
8	fatigue/tiredness	77%	fatigue, v.c.	fatigue, sleepiness, v.c.	fatigue, v.c.	fatigue, v.c.	fatigue, v.c.	fatigue, v.c.
9	orthostatism	76%						
10	brain fog	76%						
11	interruption of night sleep	75%	insomnia, v.c.					
12	weakness	74%	asthenia, uc.		asthenia, c.	asthenia, muscular weakness, uc.		
13	perceptible heartbeat	73%	palpitations, a.-r.					
14	post-exertional malaise	71%	fatigue, asthenia, v.c.	fatigue, sleepiness, v.c.	fatigue, asthenia, malaise, v.c.	fatigue, asthenia, muscular weakness, v.c., uc.	fatigue, malaise, v.c.	fatigue, v.c.
15	fasciculation	71%				tremor, uc.		
16	anxiety	69%		irritability/crying, v.c.				
17	tachycardia	66%	tachycardia, a.-r.					
18	impairment of short-term memory	65%						
19	hypersensitivity to noise	65%						
20	sleep-onset insomnia	64%	insomnia, uc.					
21	neck pain	64%	myalgia, v.c.	myalgia, v.c.	myalgia, v.c.	myalgia, v.c.	myalgia, v.c.	myalgia, muscle spasms, v.c., uc.
22	diffuse headache	63%	headache, v.c.	headache, v.c.	headache, v.c.	headache, v.c.	headache, v.c.	headache, v.c.
23	peripheral numbness	63%	hypoesthesia, n.k.	hypoesthesia, r.	hypoesthesia, uc.	hypoesthesia, uc.	hypoesthesia, n.k.	hypoesthesia, uc.
24	amnestic aphasia/anomia	61%						
25	joint pain	61%	arthralgia, v.c.	arthralgia, v.c.	arthralgia, v.c.	arthralgia, v.c.	arthralgia, v.c.	arthralgia, uc.
26	sight disorder/vision impairment	60%						
27	stress dyspnea	60%	hyperventilation, a.-r.					
28	palpitation	59%	palpitations, a.-r.					
29	sensing of internal vibrations	58%				tremor, uc.		
30	lightheadedness	58%	dizziness, uc.	dizziness, uc.	dizziness, c.	dizziness, uc.		dizziness, uc.

^1^ Complete list see Table S1. ^2^ PACVS-associated symptoms and prevalence as listed by running No. and clear name in the clinical cohort study [14]. ^3^ Adverse events as named in product information of the COVID-19-vaccines authorized for use in Europe by the EMA [16,17,18,19,20,21]; several symptoms or diagnoses are attributed if appropriate; reported frequencies of recordings abbreviated as: v.c., very common; c., common; uc., uncommon; r., rare; v.r., very rare; n.k., not known; a.-r., anxiety-related.

**Table 2:** PACVS-associated symptoms ^1^ corresponding to reactogenicity adverse events reported by the clinical phase III study of Comirnaty ^2^.

No ^3^	PACVS-Associated Symptoms ^3^	Prevalence ^3^	Monitored Adverse Events According to the Vaccines’ Product Informations ^4^					
			Biontech/Pfizer	Moderna	AstraZeneca	Janssen	Novavax	Valneva
1	exhaustion	85%	fatigue, v.c.	fatigue, sleepiness, v.c.	fatigue, v.c.	fatigue, v.c.	fatigue, v.c.	fatigue, v.c.
3	muscle pain	81%	myalgia, v.c.	myalgia, v.c.	myalgia, v.c.	myalgia, v.c.	myalgia, v.c.	myalgia, muscle spasms, v.c., uc.
8	fatigue/tiredness	77%	fatigue, v.c.	fatigue, sleepiness, v.c.	fatigue, v.c.	fatigue, v.c.	fatigue, v.c.	fatigue, v.c.
22	diffuse headache	63%	headache, v.c.	headache, v.c.	headache, v.c.	headache, v.c.	headache, v.c.	headache, v.c.
25	joint pain	61%	arthralgia, v.c.	arthralgia, v.c.	arthralgia, v.c.	arthralgia, v.c.	arthralgia, v.c.	arthralgia, uc.
50	freezing	40%	chills, v.c.	chills, v.c.	chills, v.c.	chills, c.	chills, uc.	
51	diarrhea	40%	diarrhea, v.c.	diarrhea, c.	diarrhea, c.	diarrhea, uc.		diarrhea, uc.
87	fever	20%	pyrexia, v.c.	pyrexia, v.c.	fever, v.c.		pyrexia, c.	pyrexia, c.
100	vomiting	13%	vomiting, c.	vomiting, v.c.	vomiting, c.	vomiting, uc.	vomiting, v.c.	vomiting, v.c.

^1^ Complete list see Table S1. ^2^ Reported in [22]. ^3^ PACVS-associated symptoms and prevalences as listed by running No. and clear name in the clinical cohort study [14]. ^4^ Adverse events as named in product information of the COVID-19-vaccines authorized for use in Europe by the EMA [16,17,18,19,20,21]; several symptoms or diagnoses are attributed if appropriate; reported frequencies of recordings abbreviated as: v.c., very common; c., common; uc., uncommon; r., rare; v.r., very rare; n.k., not known; a.-r., anxiety-related.

**Table 3:** PACVS-associated cardiovascular symptoms ^1^ corresponding to adverse events listed as anxiety-related in the product information of Comirnaty.

No ^2^	PACVS-Associated Symptoms ^2^	Prevalence ^2^	Monitored Adverse Events According to Product Information Sheets ^3^					
			Biontech/Pfizer	Moderna	AstraZeneca	Janssen	Novavax	Valneva
5	dizziness	80%	dizziness, uc	dizziness, uc.	Dizziness, c.	dizziness, uc.		Dizziness, uc.
13	perceptible heartbeat	73%	palpitations, a.-r.					
17	tachycardia	66%	tachycardia, a.-r.					
28	palpitation	59%	palpitations, a.-r.					
30	lightheadedness	58%	dizziness, uc.	Dizziness, uc.	Dizziness, c.	dizziness, uc.		Dizziness, uc.
31	resting tachycardia	57%	tachycardia, a.-r.					
47	cardiac arrythmia	43%	cardiac arrythmia, a.-r.					
62	hypertension	35%	blood pressure abnormalities, a.-r.				hypertension, uc.	
76	hypotension	26%	blood pressure abnormalities, a.-r.					
92	myocarditis/ pericarditis	19%	myocarditis/ pericarditis, v.r.	myocarditis/ pericarditis, v.r.		myocarditis/pericarditis, n.k.	myocarditis/pericarditis, n.k.	

^1^ Complete list see Table S1. ^2^ PACVS-associated symptoms and prevalences as listed by running number and clear name in the clinical cohort study [14]. ^3^ Adverse events as named in product information of the COVID-19-vaccines authorized for use in Europe by the EMA [16,17,18,19,20,21]; several symptoms or diagnoses are attributed if appropriate; reported frequencies of recordings abbreviated as: v.c., very common; c., common; uc., uncommon; r., rare; v.r., very rare; n.k., not known; a.-r., anxiety-related.

## Data Availability

Not applicable, no new data were created.

## Supplementary Materials

The following supporting information are available at https://www.mdpi.com/article/10.3390/vaccines12121378/s1: Table S1: Complete list of PACVS associated clinical symptoms and corresponding adverse events listed in the vaccines’ product information.

## List of Abbreviations

ACVS	acute post COVID-vaccination syndrome
EMA	European Medicines Agency
EudraVigilance	European agency monitoring post-authorization adverse drug events
FDA	Food and Drug Administration (USA)
Lareb	The Netherlands Pharmacovigilance Centre
MCAS	mast cell activation syndrome
ME/CFS	myalgic encephalomyelitis/chronic fatigue syndrome
NIH	National Institute of Health (USA)
PACS	post-acute COVID-19-syndrome (vulgo long COVID)
PACVS	post-acute post COVID-19-vaccination syndrome
POTS	postural orthostatic tachycardia syndrome
SFN	small fibre neuropathy
WHO	World Health Organisation

## References

[1] Soriano J.B., Murthy S., Marshall J.C., Relan P., Diaz J.V., WHO Clinical Case Definition Working Group on Post-COVID-19 Condition (2022). A clinical case definition of post-COVID-19 condition by a Delphi consensus. Lancet Infect Dis..

[2] Scholkmann F., May C.-A. (2023). COVID-19, post-acute COVID-19 syndrome (PACS, “long COVID”) and post-COVID-19 vaccination syndrome (PCVS, “post-COVIDvacsyndrome”): Similarities and differences. Pathol. Res. Pract..

[3] EMA Recommends First COVID-19 Vaccine for Authorisation in the EU. https://www.ema.europa.eu/en/news/ema-recommends-first-covid-19-vaccine-authorisation-eu.

[4] React19 Patient-Led Research: Persistent Symptoms Survey #1. https://react19.org/science-and-research/lit-reviews-and-surveys/react19-patient-led-research-persistent-symptoms-survey-1.

[5] Woman with ‘Life-Altering’ Injuries After COVID Vaccine Teams up with U.S. Senators to Demand Answers. https://childrenshealthdefense.org/defender/brianne-dressen-injuries-astrazeneca-covid-vaccine-senators-demand-answer/.

[6] Physician ‘Horribly Injured’ After Pfizer Vaccine Pleads with Top U.S. Public Health Officials for Help—and Gets None. https://childrenshealthdefense.org/defender/dr-danice-hertz-injured-pfizer-covid-vaccine/.

[7] Woman Injured by COVID Vaccine Pleads with Health Agencies for Help, as Local News Agency Kills Story After Pressure From Pfizer. https://childrenshealthdefense.org/defender/kristi-dobbs-injured-pfizer-covid-vaccine-local-news-agency-kills-story/.

[8] Couzin-Frankel J., Vogel G. (2022). In rare cases, coronavirus vaccines may cause Long Covid–like symptoms. Sci. Insid..

[9] Mandavilli A. (2024). Thousands Believe COVID Vaccines Harmed Them. Is Anyone Listening?. New York Times.

[10] Safavi F., Gustafson L., Walitt B., Lehky T., Dehbashi S., Wiebold A., Mina Y., Shin S., Pan B., Polydefkis M. (2022). Neuropathic symptoms with SARS-CoV-2 vaccination. medRxiv [Preprint].

[11] European Medicines Agency, COVID-19 Vaccines Safety Update, November 2022 Rev. 3. https://www.ema.europa.eu/en/documents/covid-19-vaccine-safety-update/covid-19-vaccines-safety-update-10-november-2022_en.pdf.

[12] Semmler A., Mundorf A.K., Kuechler A.S., Schulze-Bosse K., Heidecke H., Schulze-Forster K., Schott M., Uhrberg M., Weinhold S., Lackner K.J. (2023). Chronic Fatigue and Dysautonomia following COVID-19 Vaccination Is Distinguished from Normal Vaccination Response by Altered Blood Markers. Vaccines.

[13] Krumholz H.M., Wu Y., Sawano M., Shah R., Zhou T., Arun A.S., Khosla P., Kaleem S., Vashist A., Bhattacharjee B. (2023). Post-Vaccination Syndrome: A Descriptive Analysis of Reported Symptoms and Patient Experiences After Covid-19 Immunization. medRxiv [Preprint].

[14] Mundorf A.K., Semmler A., Heidecke H., Schott M., Steffen F., Bittner S., Lackner K.J., Schulze-Bosse K., Pawlitzki M., Meuth S.G. (2024). Clinical and Diagnostic Features of Post-Acute COVID-19 Vaccination Syndrome (PACVS). Vaccines.

[15] EudraVigilance. https://www.ema.europa.eu/en/human-regulatory-overview/research-development/pharmacovigilance-research-development/eudravigilance.

[16] EPAR Product Information Sheets, Annex 1, Summary of Product Characteristics. https://www.ema.europa.eu/en/documents/product-information/comirnaty-epar-product-information_en.pdf.

[17] EPAR Product Information Sheets, Annex 1, Summary of Product Characteristics. https://www.ema.europa.eu/en/documents/product-information/spikevax-epar-product-information_en.pdf.

[18] EPAR Product Information Sheets, Annex 1, Summary of Product Characteristics. https://www.ema.europa.eu/en/documents/product-information/vaxzevria-epar-product-information_en.pdf.

[19] EPAR Product Information Sheets, Annex 1, Summary of Product Characteristics. https://www.ema.europa.eu/en/documents/product-information/jcovden-epar-product-information_en.pdf.

[20] EPAR Product Information Sheets, Annex 1, Summary of Product Characteristics. https://www.ema.europa.eu/en/documents/product-information/nuvaxovid-epar-product-information_en.pdf.

[21] EPAR Product Information Sheets, Annex 1, Summary of Product Characteristics. https://www.ema.europa.eu/en/documents/product-information/covid-19-vaccine-inactivated-adjuvanted-valneva-epar-product-information_en.pdf.

[22] Polack F.P., Thomas S.J., Kitchin N., Absalon J., Gurtman A., Lockhart S., Perez J.L., Marc G.P., Moreira E.D., Zerbini C. (2020). Safety and Efficacy of the BNT162b2 mRNA Covid-19 Vaccine. N. Engl. J. Med..

[23] Adverse Event Monitoring Browse Under the Letter “C” LIKE Covid-19 Vaccines and the Respective Vaccine Brand. https://www.adrreports.eu/en/search_subst.html.

[24] EudraVigilance Access Policy. https://www.ema.europa.eu/en/documents/other/european-medicines-agency-policy-access-eudravigilance-data-medicinal-products-human-use-revision-4_en.pdf.

[25] Riad A., Pokorná A., Attia S., Klugarová J., Koščík M., Klugar M. (2021). Prevalence of COVID-19 Vaccine Side Effects among Healthcare Workers in the Czech Republic. J. Clin. Med..

[26] COVID-19 Vaccine Safety Update, COMIRNATY BioNTech Manufacturing GmbH, 06. October 2021. https://www.ema.europa.eu/en/documents/covid-19-vaccine-safety-update/covid-19-vaccine-safety-update-comirnaty-6-october-2021_en.pdf.

[27] COMIRNATY Procedural Steps Taken and Scientific Information After the Authorisation. https://www.ema.europa.eu/en/documents/procedural-steps-after/comirnaty-epar-procedural-steps-taken-scientific-information-after-authorisation_en.pdf.

[28] Centrum Lareb: Long COVID-Like Symptoms Following Immunization with COVID-19 Vaccines. https://www.lareb.nl/media/g54d3xrc/signals_2024_long-covid-like-symptoms-after-covid-19-vaccination_website.pdf.

[29] Finsterer J. (2024). Myocarditis, Coagulopathy, and Small Fibre, Sensory, and Multiple Cranial Nerve Neuropathy Complicating BNT162b2 Vaccination: A Case Report. Cureus.

[30] Assessment Report EMA/707383/2020 Corr. 2. (2021). https://www.ema.europa.eu/en/documents/assessment-report/comirnaty-epar-public-assessment-report_en.pdf.

[31] Speicher D.J., Rose J., Gutschi L.M., Wiseman D.M., McKernan K. (2023). DNA fragments detected in monovalent and bivalent Pfizer/BioNTech and Moderna modRNA COVID-19 vaccines from Ontario, Canada: Exploratory dose response relationship with serious adverse events. Researchgate [Preprint].

[32] South Carolina Legislature Online, 12 September 2023. https://www.scstatehouse.gov/CommitteeInfo/SenateMedicalAffairsCommittee/PandemicPreparedness/Phillip-Buckhaults-SC-Senate-09122023-final.pdf.

[33] König B., Kirchner J.O. (2024). Methodological Considerations Regarding the Quantification of DNA Impurities in the COVID-19 mRNA Vaccine Comirnaty®. Methods Protoc..

[34] Information for Healthcare Professionals, 22 December 2023. https://www.pei.de/SharedDocs/Downloads/EN/newsroom-en/notification/231222-testing-mrna-vaccinas-dna-contamination.pdf.

[35] Kulkarni J.A., Myhre J.L., Chen S., Tam Y.Y.C., Danescu A., Richman J.M., Cullis P.R. (2017). Design of lipid nanoparticles for in vitro and in vivo delivery of plasmid DNA. Nanomedicine.

[36] Theoharides T.C. (2022). Could SARS-CoV-2 Spike Protein Be Responsible for Long-COVID Syndrome?. Mol. Neurobiol..

[37] OECD, Enhancing Public Trust in COVID-19 Vaccination. https://one.oecd.org/document/COM/DELSA/GOV(2021)1/En/pdf.

[38] Gopalaswamy R., Aravindhan V., Subbian S. (2024). The Ambivalence of Post COVID-19 Vaccination Responses in Humans. Biomolecules.

[39] Thoma M., Froehlich L., Hattesohl D.B.R., Quante S., Jason L.A., Scheibenbogen C. (2023). Why the Psychosomatic View on Myalgic Encephalomyelitis/Chronic Fatigue Syndrome Is Inconsistent with Current Evidence and Harmful to Patients. Medicina.

[40] Valent P., Hartmann K., Bonadonna P., Gülen T., Brockow K., Alvarez-Twose I., Hermine O., Niedoszytko M., Carter M.C., Hoermann G. (2022). Global Classification of Mast Cell Activation Disorders: An ICD-10-CM-Adjusted Proposal of the ECNM-AIM Consortium. J. Allergy Clin. Immunol. Pract..

[41] Raj V., Opie M., Arnold A.C. (2018). Cognitive and psychological issues in postural tachycardia syndrome. Auton Neurosci.

[42] Fabry V., Gerdelat A., Acket B., Cintas P., Rousseau V., Uro-Coste E., Evrard S.M., Pavy-Le Traon A. (2020). Which Method for Diagnosing Small Fiber Neuropathy?. Front Neurol..

[43] Geerts M., Hoeijmakers J.G.J., Gorissen-Brouwers C.M.L., Faber C.G., Merkies I.S.J. (2023). Small Fiber Neuropathy: A Clinical and Practical Approach. J. Nurse Pract..

[44] Mastropaolo M., Hasbani M.J. (2023). Small Fiber Neuropathy Triggered by COVID-19 Vaccination: Association with FGFR3 Autoantibodies and Improvement during Intravenous Immunoglobulin Treatment. Case Rep. Neurol..

[45] Long-COVID Richtlinie. https://www.g-ba.de/downloads/62-492-3451/LongCOV-RL_2023-12-21_iK-2024-05-09.pdf.

[46] Buonsenso D., Tantisira K.G. (2024). Long COVID and SARS-CoV-2 persistence: New answers, more questions. Lancet Infect Dis..

[47] Davis H.E., McCorkell L., Vogel J.M., Topol E.J. (2023). Long COVID: Major findings, mechanisms and recommendations. Nat. Rev. Microbiol..

[48] Peluso M.J., Deeks S.G. (2024). Mechanisms of long COVID and the path toward therapeutics. Cell.

[49] Leng A., Shah M., Ahmad S.A., Premraj L., Wildi K., Li Bassi G., Pardo C.A., Choi A., Cho S.M. (2023). Pathogenesis Underlying Neurological Manifestations of Long COVID Syndrome and Potential Therapeutics. Cells.

[50] Cancarevic I., Nassar M., Medina L., Sanchez A., Parikh A., Hosna A., Devanabanda B., Vest M., Ayotunde F., Ghallab M. (2022). Nephrotic Syndrome in Adult Patients With COVID-19 Infection or Post COVID-19 Vaccine: A Systematic Review. Cureus.

[51] Chen Y., Xu Z., Wang P., Li X.M., Shuai Z.W., Ye D.Q., Pan H.F. (2022). New-onset autoimmune phenomena post-COVID-19 vaccination. Immunology.

[52] Finsterer J., Scorza F.A., Scorza C.A. (2021). Post SARS-CoV-2 vaccination Guillain-Barre syndrome in 19 patients. Clinics.

[53] Abolmaali M., Rezania F., Behnagh A.K., Hamidabad N.M., Gorji A., Mirzaasgari Z. (2022). Guillain-Barré syndrome in association with COVID-19 vaccination: A systematic review. Immunol. Res..

