# Supplementary material for: The Post-Acute COVID-19-Vaccination Syndrome in the Light of Pharmacovigilance
Source: Vaccines (Basel). 2024 Dec 6;12(12):1378. doi: 10.3390/vaccines12121378 (PMC11680367; doi:10.3390/vaccines12121378)
Supplement: Supplementary file 1 [file vaccines-12-01378-s001.zip › vaccines-3315094-supplementary.pdf]

**Table S1: PACVS associated clinical symptoms and corresponding adverse events listed in the vaccines' product informations**

| No[1] | PACVS-associated symptoms[1] | Prevalence[1] | monitored adverse events according to product information sheets[2] | | | | | |
|---|---|---|---|---|---|---|---|---|
| | | | Biontech /Pfizer | Moderna | AstraZeneca | Janssen | Novavax | Valneva |
| 1 | exhaustion | 85% | fatigue, v.c. | fatigue, sleepiness, v.c. | fatigue, v.c. | fatigue, v.c. | fatigue, v.c. | fatigue, v.c. |
| 2 | debility | 84% | asthenia, uc | | asthenia, c. | asthenia, muscular weakness, uc. | | |
| 3 | muscle pain | 81% | myalgia, v.c. | myalgia, v.c. | myalgia, v.c. | myalgia, v.c. | myalgia, v.c. | myalgia, muscle spasms, v.c., uc. |
| 4 | unrestful sleep | 81% | insomnia, uc. | | | | | |
| 5 | dizziness | 80% | dizziness, uc. | dizziness, uc. | dizziness, c. | dizziness, uc. | | dizziness, uc. |
| 6 | tingling/prickling/paresthesia | 80% | paresthesia, n.k. | paresthesia, r. | paresthesia, uc. | paresthesia, r. | paresthesia, n.k. | paresthesia, uc. |
| 7 | impairment of mental focussing | 79% | | | | | | |
| 8 | fatigue / tiredness | 77% | fatigue, v.c. | fatigue, sleepiness, v.c. | fatigue, v.c. | fatigue, v.c. | fatigue, v.c. | fatigue, v.c. |
| 9 | orthostatism | 76% | | | | | | |
| 10 | brain fog | 76% | | | | | | |
| 11 | interruption of night sleep | 75% | insomnia, v.c. | | | | | |
| 12 | weakness | 74% | asthenia, uc. | | asthenia, c. | asthenia, muscular weakness, uc. | | |

| No[1] | PACVS-associated symptoms[1] | Prevalence[1] | monitored adverse events according to product information sheets[2] | | | | | |
|---|---|---|---|---|---|---|---|---|
| | | | Biontech /Pfizer | Moderna | AstraZeneca | Janssen | Novavax | Valneva |
| 13 | perceptible heartbeat | 73% | palpitations, a.-r. | | | | | |
| 14 | post-exertional malaise | 71% | fatigue, asthenia, v.c. | fatigue, sleepiness, v.c. | fatigue, asthenia, malaise, v.c. | fatigue, asthenia, muscular weakness, v.c., uc. | fatigue, malaise, v.c. | fatigue, v.c. |
| 15 | fasciculation | 71% | | | | tremor, uc. | | |
| 16 | anxiety | 69% | | irritability / crying, v.c. | | | | |
| 17 | tachycardia | 66% | tachycardia, a.-r. | | | | | |
| 18 | impairment of short-term memory | 65% | | | | | | |
| 19 | hypersensitivity to noise | 65% | | | | | | |
| 20 | sleep-onset insomnia | 64% | insomnia, uc. | | | | | |
| 21 | neck pain | 64% | myalgia, v.c. | myalgia, v.c. | myalgia, v.c. | myalgia, v.c. | myalgia, v.c. | myalgia, muscle spasms, v.c., uc. |
| 22 | diffuse headache | 63% | headache, v.c. | headache, v.c. | headache, v.c. | headache, v.c. | headache, v.c. | headache, v.c. |
| 23 | peripheral numbness | 63% | hypoesthesia, n.k. | hypoesthesia, r. | hypoesthesia, uc. | hypoesthesia, uc. | hypoesthesia, n.k. | hypoesthesia, uc. |
| 24 | amnestic aphasia / anomia | 61% | | | | | | |
| 25 | joint pain | 61% | arthralgia, v.c. | arthralgia, v.c. | arthralgia, v.c. | arthralgia, v.c. | arthralgia, v.c. | arthralgia, uc. |
| 26 | sight disorder / vision impairment | 60% | | | | | | |

| No[1] | PACVS-associated symptoms[1] | Prevalence[1] | monitored adverse events according to product information sheets[2] | | | | | |
|---|---|---|---|---|---|---|---|---|
| | | | Biontech /Pfizer | Moderna | AstraZeneca | Janssen | Novavax | Valneva |
| 27 | stress dyspnea | 60% | hyperventilation, a.-r. | | | | | |
| 28 | palpitation | 59% | palpitations, a.-r. | | | | | |
| 29 | sensing of internal vibrations | 58% | | | | tremor, uc. | | |
| 30 | lightheadedness | 58% | dizziness, uc. | dizziness, uc. | dizziness, c. | dizziness, uc. | | dizziness, uc. |
| 31 | resting tachycardia | 57% | tachycardia, a.-r. | | | | | |
| 32 | sensing electrical current flow through body | 56% | | | | | | |
| 33 | impairment of ocular focussing | 56% | | | | | | |
| 34 | hypersensitivity to light | 56% | | | | | | photophobia, r. |
| 35 | weight change more than 5 kg | 54% | | | | | | |
| 36 | nausea | 52% | nausea, c. | nausea, v.c. | nausea, v.c. | nausea, v.c. | nausea, v.c. | nausea, v.c. |
| 37 | sicca syndrome | 50% | | | | | | |
| 38 | tinnitus | 48% | | | tinnitus, uc. | tinnitus, r. | | |
| 39 | Post-vaccine syndrome | 48% | no adverse event detection possible | | | | | |
| 40 | nocturnal sweating | 47% | night sweats, uc. | | | | | |
| 41 | angina pectoris | 46% | | | | | | |
| 42 | burning hand / feet | 46% | paresthesia, n.k. | paresthesia, r. | paresthesia, uc. | paresthesia, r. | paresthesia, n.k. | paresthesia, uc. |
| 43 | histamine intolerance | 45% | | | | | | |

| No[1] | PACVS-associated symptoms[1] | Prevalence[1] | monitored adverse events according to product information sheets[2] | | | | | |
|---|---|---|---|---|---|---|---|---|
| | | | Biontech /Pfizer | Moderna | AstraZeneca | Janssen | Novavax | Valneva |
| 44 | retro-orbital pain | 44% | headache, v.c. | headache, v.c. | headache, v.c. | headache, v.c. | headache, v.c. | headache, v.c. |
| 45 | appetite loss | 43% | decreased appetite, uc. | | decreased appetite, uc. | | | |
| 46 | panic attacks | 43% | a.-r. | | | | | |
| 47 | cardiac arrythmia | 43% | cardiac arrythmia, a.-r. | | | | | |
| 48 | abnormal estrous cycle | 43% | heavy menstrual bleeding, n.k. | heavy menstrual bleeding, n.k. | | | | |
| 49 | disturbed / altered temperature perception | 41% | | | | | | |
| 50 | freezing | 40% | chills, v.c. | chills, v.c. | chills, v.c. | chills, c. | chills, uc. | |
| 51 | diarrhea | 40% | diarrhea, v.c. | diarrhea, c. | diarrhea, c. | diarrhea, uc. | | diarrhea, uc. |
| 52 | genereal feeling of illness | 39% | no adverse event detection possible | | | | | |
| 53 | resting dyspnea | 39% | | | | | | |
| 54 | adrenal hits / unmotivated stress response | 39% | a.-r. | | | | | |
| 55 | helmet sensation / head constriction | 39% | headaches, v.c. | headaches, v.c. | headaches, v.c. | headaches, v.c. | headache, v.c. | headache, v.c. |
| 56 | lymphe node swelling | 39% | lymphadenopathy, c. | | lymphadenopathy, uc. | lymphadenopathy, r. | lymphadenopathy, uc. | lymphadenopathy, uc. |
| 57 | visible / prominent veins | 38% | | | | | | |
| 58 | polyuria | 37% | | | | | | |
| 59 | hair loss | 37% | | | | | | |
| 60 | insomnia | 36% | insomnia, uc. | | | | | |

| No[1] | PACVS-associated symptoms[1] | Prevalence[1] | monitored adverse events according to product information sheets[2] | | | | | |
|---|---|---|---|---|---|---|---|---|
| | | | Biontech /Pfizer | Moderna | AstraZeneca | Janssen | Novavax | Valneva |
| 61 | long-Covid without Covid infection | 36% | no adverse event detection possible | | | | | |
| 62 | hypertension | 35% | blood pressure abnormalities, a.-r. | | | | hypertension, uc. | |
| 63 | tremor | 35% | | | | tremor, uc. | | |
| 64 | depression | 33% | | irritability / crying, v.c. | | | | |
| 65 | shivering | 32% | chills, v.c. | chills, v.c. | chills, v.c. | chills, v.c. | | |
| 66 | polydipsia | 32% | | | | | | |
| 67 | ME/CFS | 32% | fatigue, v.c. | fatigue, sleepiness, v.c. | fatigue, asthenia, malaise, v.c. | fatigue, asthenia, muscular weakness, v.c., uc. | fatigue, malaise, v.c. | fatigue, v.c. |
| 68 | paralysis | 31% | | acute peripheral facial paralysis, r. | facial paralysis, Guillain-Barré syndrome, r., v.r. | facial paralysis, Guillain-Barré syndrome, r., v.r | | |
| 69 | migraine | 30% | headache, v.c. | headache, v.c. | headache, v.c. | headache, v.c. | haedache, v.c. | migraine, uc. |
| 70 | allergy | 30% | hypersensitivity reactions, uc. | | hypersensitivity, n.k. | hypersensitivity, r. | | |
| 71 | stiffness of joints | 29% | arthralgia, v.c. | arthralgia, v.c. | arthralgia, v.c. | arthralgia, uc. | arthralgia, v.c. | arthralgia, uc. |
| 72 | itching | 29% | pruritus, uc. | | pruritus, uc. | | pruritus, uc. | |
| 73 | irritatable colon | 28% | diarrhea, v.c. | diarrhea, abdominal pain, c., uc. | diarrhea, c. | diarrhea, uc. | | |

| No[1] | PACVS-associated symptoms[1] | Prevalence[1] | monitored adverse events according to product information sheets[2] | | | | | |
|---|---|---|---|---|---|---|---|---|
| | | | Biontech /Pfizer | Moderna | AstraZeneca | Janssen | Novavax | Valneva |
| 74 | spontaneous bruising | 28% | | | | | | |
| 75 | reactivation of infections | 27% | | | | | | |
| 76 | hypotension | 26% | a.-r. | | | | | |
| 77 | rash | 26% | rash, uc. | rash, c. | rash, uc. | | rash, uc. | rash, uc. |
| 78 | aphthae | 25% | | | | | | |
| 79 | goosebumps | 24% | chills, v.c. | chills, v.c. | chills, v.c. | chills, v.c. | chills, v.c. | |
| 80 | vaccine-induced inflammation response | 24% | | | | | | |
| 81 | petechiae | 23% | | | | | | |
| 82 | hypoventilation | 22% | | | | | | |
| 83 | Herpes labialis | 21% | | | | | | |
| 84 | POTS | 21% | | | | | | |
| 85 | inflammed joints | 20% | arthralgia, v.c. | arthralgia, v.c. | arthralgia, v.c. | arthralgia, uc. | arthralgia, v.c. | arthralgia, uc. |
| 86 | flush | 20% | hypersensitivity reactions, uc. | | | | | |
| 87 | fever | 20% | pyrexia, v.c. | pyrexia, v.c. | fever, v.c. | | pyrexia, c. | pyrexia, c. |
| 88 | acrocyanosis | 20% | | | | | | |
| 89 | reduced nerve conduction velocity | 20% | | | | | | |
| 90 | dysphagia | 20% | | | | | | |
| 91 | MCAS | 19% | | | | | | |
| 92 | myocarditis / pericarditis | 19% | myocarditis / pericarditis, v.r. | myocarditis / pericarditis, v.r. | | myocarditis / pericarditis, n.k. | myocarditis / pericarditis, n.k. | |
| 93 | fibromyalgia | 17% | myalgia, v.c. | myalgia, v.c. | myalgia, v.c. | myalgia, v.c. | myalgia, v.c. | myalgia, v.c. |

| No[1] | PACVS-associated symptoms[1] | Prevalence[1] | monitored adverse events according to product information sheets[2] | | | | | |
|---|---|---|---|---|---|---|---|---|
| | | | Biontech /Pfizer | Moderna | AstraZeneca | Janssen | Novavax | Valneva |
| 94 | SFN | 17% | paresthesia / hypoesthesia, n.k. | paresthesia / hypoesthesia, r. | paresthesia / hypoesthesia, uc. | paresthesia / hypoesthesia, r. | paresthesia / hypoesthesia, n.k. | paresthesia / hypoesthesia, uc. |
| 95 | constipation | 17% | | | | | | |
| 96 | edema | 14% | angioedema, uc. | | | | | |
| 97 | bradycardia | 14% | | | | | | |
| 98 | disturbed breathing excursion | 14% | | | | | | |
| 99 | mycosis | 13% | | | | | | |
| 100 | vomiting | 13% | vomiting, c. | vomiting, v.c. | vomiting, c. | vomiting, uc. | vomiting, v.c. | vomiting,v.c. |
| 101 | sleep apnea | 12% | | | | | | |
| 102 | sleep paralysis | 8% | | | | | | |
| 103 | hyperventilation | 8% | a.-r. | | | | | |
| 104 | hemangioma | 7% | | | | | | |
| 105 | thrombosis | 7% | | | venous thromboembolism, n.k. | thrombosis in combination with thrombocytopenia, v.r. | | thrombophlebitis, r. |
| 106 | anaphylactic shock | 6% | anaphylaxis, n.k. | | anaphylaxis, n.k. | anaphylaxis, n.k. | anaphylaxis, n.k. | |
| 107 | shingles | 6% | | | | | | |
| 108 | Guillain-Barre syndrome | 5% | | | Guillain-Barré syndrome, v.r. | Guillain-Barré syndrome, v.r. | | |
| 109 | emphysema | 4% | | | | | | |

| No[1] | PACVS-associated symptoms[1] | Prevalence[1] | monitored adverse events according to product information sheets[2] | | | | | |
|---|---|---|---|---|---|---|---|---|
| | | | Biontech /Pfizer | Moderna | AstraZeneca | Janssen | Novavax | Valneva |
| 110 | facial nerve paresis | 4% | acute peripheral facial paralysis, r. | acute peripheral facial paralysis, r. | facial paralysis, r. | facial paralysis, r. | | |

[1]PACVS-associated symptoms and prevalences as listed by running number and clear name in the clinical cohort study [14]

[2]Adverse events as named in product information of the vaccines [16]; several symptoms or diagnoses are attributed if appropriate; reported frequencies of recordings abbreviated as: v.c., very common; c., common; uc., uncommon; r., rare; v.r.,very rare; n.k.,not known; a.-r., anxiety-related.
